# A Hybrid Model with New Word Weighting for Fast Filtering Spam Short Texts

**DOI:** 10.3390/s23218975

**Published:** 2023-11-04

**Authors:** Tian Xia, Xuemin Chen, Jiacun Wang, Feng Qiu

**Affiliations:** 1 School of Computer and Information Engineering, Shanghai Polytechnic University, Shanghai 201209, China; xiatian@sspu.edu.cn; 2 Department of Engineering, Texas Southern University, Houston, TX 77004, USA; xuemin.chen@tsu.edu; 3 Department of Computer Science and Software Engineering, Monmouth University, West Long Branch, NJ 07764, USA; jwang@monmouth.edu; 4 Institute of Artificial Intelligence on Education, Shanghai Normal University, Shanghai 200234, China

**Keywords:** natural language processing, text classification, short text, new word weighting, artificial neural network, hidden Markov model

## Abstract

Short message services (SMS), microblogging tools, instant message apps, and commercial websites produce numerous short text messages every day. These short text messages are usually guaranteed to reach mass audience with low cost. Spammers take advantage of short texts by sending bulk malicious or unwanted messages. Short texts are difficult to classify because of their shortness, sparsity, rapidness, and informal writing. The effectiveness of the hidden Markov model (HMM) for short text classification has been illustrated in our previous study. However, the HMM has limited capability to handle new words, which are mostly generated by informal writing. In this paper, a hybrid model is proposed to address the informal writing issue by weighting new words for fast short text filtering with high accuracy. The hybrid model consists of an artificial neural network (ANN) and an HMM, which are used for new word weighting and spam filtering, respectively. The weight of a new word is calculated based on the weights of its neighbor, along with the spam and ham (i.e., not spam) probabilities of short text message predicted by the ANN. Performance evaluations on benchmark datasets, including the SMS message data maintained by University of California, Irvine; the movie reviews, and the customer reviews are conducted. The hybrid model operates at a significantly higher speed than deep learning models. The experiment results show that the proposed hybrid model outperforms other prominent machine learning algorithms, achieving a good balance between filtering throughput and accuracy.

## 1. Introduction

Nowadays, a multitude of short texts are generated through various communication channels, such as short message services (SMS), microblogging, instant messaging services, e-commercial services. For instance, Twitter receives approximately 6000 posts per second [1]. Short texts serve as a convenient and cost-effective means to connect with individuals. Research indicates the high reliability of SMS, with 99% of all SMS messages being read by their recipients [2]. For this reason, spammers take advantage of short texts to spread unwanted advertisements and malicious messages.

The detection for short texts presents unique difficulties because of their characteristics [3]. First, the limited length may not contain sufficient semantic information. Second, a wide range of topics results in a high degree of sparsity within the short text representation matrix [4]. Third, short texts are rapidly and constantly generated, necessitating real-time, high-throughput spam filtering. Lastly, informal writing is prevalent in short texts. In other words, short texts are frequently composed in a casual, informal, idiosyncratic, and occasionally misspelled manner [5]. For example, people commonly substitute “thx” for “thanks”, and “im here” for “I am here”. Some short texts are even intentionally and maliciously crafted to evade spam filters [6]. For instance, spammers may employ “kredit kard” for “credit card”, and “banc acct” for “bank account”. This informal writing style introduces numerous new words to short texts, thereby complicating the identification of spam messages.

Researchers have invested significant efforts in short text spam filtering. Traditional learning methods, including statistical techniques such as naïve Bayes (NB) [7], the vector space model (VSM) [8], the support vector machine (SVM) [9], and k-nearest neighbor (KNN) [10], often treat text as a collection of independent words and disregard word order. These approaches rely on statistical feature extraction methods like TF-IDF (term frequency-inverse document frequency) [11]. To further improve accuracy, deep learning models have recently been deployed to address these issues. In addressing the challenge of limited text length, often referred to as the “shortness issue,” several approaches have been implemented. Gao et al. [4] addressed this issue by implementing a convolutional neural network (CNN) and a bidirectional gated recurrent units model (Bi-GRU), seamlessly integrated with the TF-IDF algorithm. Zhu et al. [12] harnessed the power of bidirectional encoder representation from transformers (BERT) to extract more relevant features from the user’s sentiment context. Machicao et al. [13] proposed a novel approach that combines network structure and dynamics based on cellular automata theory to capture patterns in text networks and thus enhancing the text representation. The latest research, building upon the advancements of the generative pre-trained transformer 3 (GPT-3), achieves even higher accuracy [14]. To address the issue of sparsity, researchers have adopted strategies to expand and enrich the feature space. Liao et al. [15] treated each category as a subtask and utilized the robustly optimized BERT pre-training method, based on the deep bidirectional Transformer, to extract features from both the text and category tokens. Wang et al. [16] addressed sparsity by semantically expanding short texts. They incorporated an attention mechanism into their neural network model to identify and include related words from the short text. Cai et al. [17] used the attention mechanism to further strengthen the extraction of sentiment features. However, the deep learning models can achieve high accuracy by complex architectures, which demands heavy computation. Nevertheless, it is important to note that the deep learning approaches prioritize achieving higher accuracy and may overlook considerations related to training and filtering speed. Most of these algorithms are still in their early stages of development [18] because balancing the objectives of achieving high accuracy and maintaining high-throughput spam filtering is a formidable challenge and is particularly critical in the context of the filtering industry. This study treats the limited words in a short text as a sequence with dependent features. Making use of word order, we applied a hidden Markov model (HMM) for short text filtering, which achieves high accuracy and high throughput.

Furthermore, it is crucial to identify the frequently occurring unknown strings because of the shortness issue. However, the research in new word weighting, particularly mitigating the informal writing issue under the specific challenges of short text filtering, is limited. Several methods have been devised to identify new words in long texts. These methods involve extracting independent features from the remaining portions of the text. Qian et al. [19] used word embedding and frequent n-grams string mining to identify new word for Chinese word segmentation. For a similar purpose, Duan et al. [20] used the bidirectional long short-term memory (LSTM) model and conditional random fields to process the manually chosen features, including word length, part of speech, contextual entropy, and degree of word coagulation.

More importantly, scaling the extent to which a new word signifies divergence holds particular significance in the field of short text filtering given the constraints of text length limitation, feature sparsity, computational resources, and performance considerations [21]. The weight assigned to a new word is linked to the presence of other known words in all short texts where it occurs. This study introduces a novel approach to calculate a new word weight based on both the weights of known words in short texts and the artificial neural network (ANN) predicted probabilities of the texts being ham or spam. The novel weighting method further improves the accuracy further without compromising processing speed, thus achieving superior performance and filtering speed concurrently.

In summary, this study proposes a hybrid model to tackle the challenges posed by informal writing in fast filtering short texts. It combines an ANN for new word weighting and an HMM for filtering at a high processing speed without imposing a heavy computational burden. Extensive experiments are conducted on the SMS Spam Collection hosted at the University of California, Irvine (UCI) and other four datasets to illustrate the effectiveness of the proposed hybrid model. The performances are evaluated in key criteria such as accuracy, training time, training speed, and filtering throughput, enabling a thorough comparison of the proposed hybrid model’s capabilities.

The contributions of the paper are summarized as follows:

- A novel new word weighting method based on the ANN model is developed. The weight of a word measures its likelihood of being densely distributed in one category. The weight of a new word in a short text is weighted based on the weights of its neighbor words and the probabilities yielded by the ANN.
- When all words are properly weighted, a hybrid model that combines the ANN and an HMM is proposed for accurate and fast short text filtering. The HMM is used to predict the likelihood of a short text being spam.
- The hybrid model represents pioneering research in the specialized domain of short text filtering, addressing unique challenges like limited length and feature sparsity with novel approaches.

The rest of the paper is organized as follows. The proposed hybrid model is presented in Section 2. The evaluation metrics, experimental results, and discussion are given in Section 3. The conclusions and future work are drawn in Section 4.

## 2. The Hybrid Model for Short Text Filtering

In this section, we describe, in detail, the short text pre-processing, feature extraction, and proposed hybrid model for fast short text filtering.

### 2.1. Short Text Pre-Processing

The short texts are first pre-processed. This involves the following processes:

- Case folding converts all capital letters in the data set into lowercase characters.
- Tokenization divides the raw text into individual words.
- Stemming and Lemmatization chop off the affixes of words and transforms them into their base form.
- Stop words removal eliminates the common words that only stand for positioning.

In addition to common stop words, we refine the list by excluding words that frequently appear in specific categories of messages. For example, we retain symbols like ‘$’ often associated with financial content, making them valuable for identifying spam. We also expand the list to include emojis and special character strings like ‘&lt’ and ‘#&gt’. This decision is guided by the recognition that these elements are commonly found in both spam and ham messages, making them less informative for classification.

### 2.2. Feature Extraction

The remaining words in pre-processed short texts have different semantic importance. A word that frequently appears in one category, whether it is ham or spam, may contain more semantic information related to that category. Inspired by this observation, the feature extraction algorithm calculates the difference in word occurrence probabilities as the weight for that word, i.e.,

(1) $$w_{i}=P\left(t_{i}\in s_{1}\right)-P\left(t_{i}\in s_{2}\right),$$

where $t_{i}$ refers to a word in pre-processed short texts, and $s_{1}$ and $s_{2}$ are the ham and spam categories, respectively. With Equation (1), negative weights are assigned to words that are indicative of spam content and positive weights to words that typically appear in ham messages. After that, the weight for each word is mapped to a number between −10 and 10 by the normalization process, i.e.,

(2) $$w_{i}^{normalized}=\left(f\left(w_{i}\right)-0.5\right)\times 20,$$

where $f\left(w_{i}\right)=\frac{1}{1+exp(-w_{i})}$ is the sigmoid function.

### 2.3. ANN for New Word Weighting

#### 2.3.1. Short Text Representation

The short text vectors and their ham or spam label pairs, (vector,label), are inputs of the ANN. The short text vectors inherit the term-document vectors in vector space model [22]. Each element of the short text vectors represents a distinct word in the pre-processed training data.

Suppose $T=\{t_{1},t_{2},\ldots ,t_{N}\}$ is the set of all distinct words in the pre-processed training set where *N* is the total number of distinct words. After feature extraction described in Section 2.2, each word has a weight, and the weights for *T* can be represented as $w_{T}=\left(w_{t_{1}},w_{t_{2}},\ldots ,w_{t_{N}}\right)$. The *k*th short text vector is denoted as $V_{k}=\left(w_{t_{1}}^{k},w_{t_{2}}^{k},w_{t_{j}}^{k},\ldots ,w_{t_{N}}^{k}\right)$, where $w_{t_{j}}^{k}$ is the weight of the *j*th word and $w_{t_{j}}^{k}=w_{t_{j}}$ if the *j*th word is present in the *k*th short text. Otherwise, $w_{t_{j}}^{k}=0$. Suppose there are *M* short texts in the training set. The short text vectors $V_{1},V_{2},\ldots ,V_{M}$ form vector space.

#### 2.3.2. Training of the ANN

The proposed ANN is a typical feedforward network. The (vector,label) pairs can be described as $V=\{\left(V_{1},y_{1}\right),$$\left(V_{2},y_{2}\right),\ldots ,\left(V_{M},y_{M}\right)\}$, where $y_{k}\in \left\{ham,spam\right\}$. Each layer in ANN uses the output of previous layer as the input. Suppose the previous layer has *n* neurons; each neuron in the subsequent layer yields an output based on the inputs $x_{j}$, synaptic weights $w_{j}^{ANN}$, and $bias_{j}$, 1≤j≤n. The output is calculated by $y=f\left(\sum_{j=1}^{n}w_{j}^{ANN}x_{j}\right)+bias_{j}$, where function *f* is the sigmoid activation function. The ANN model has three hidden layers. Each hidden layer consists of 100 neurons. The output layer has 1 neuron, indicating ham or spam label by probabilities. Define $ANN(V_{k})$ as the ANN operation applied to $V_{k}$. The probabilities are denoted as $P(ANN\left(V_{k}\right)=ham)$ and $P(ANN\left(V_{k}\right)=spam)$, where $P\left(ANN\left(V_{k}\right)=ham\right)+P\left(ANN\left(V_{k}\right)=spam\right)=1$.

The synaptic weights and the biases used in computation are initialized randomly and updated in the backward propagation algorithm according to the mean square error loss function. The training of the ANN is terminated when the value of loss function is reduced below a specified threshold.

#### 2.3.3. New Word Weighting Based on ANN Probability

When an unidentified word comes up, it is submitted to a module called the new word reporter, which maintains a list of unidentified words. Any new word is reported to the ANN for weighting when the number of the short texts where it occurs exceeds the preset threshold *q*.

The weighting process includes three steps, as shown in Algorithm 1:

In step 1, suppose a new word existed in the *q* testing short texts, which are represented as the following vectors: $\left\{V_{1}^{\prime },V_{2}^{\prime },\ldots ,V_{q}^{\prime }\right\}$. The $k^{th}$ short text vector $V_{k}^{\prime }$ consists of the weights of known words, represented as $\left({w_{t_{1}}^{k}}^{\prime },{w_{t_{2}}^{k}}^{\prime },{w_{t_{j}}^{k}}^{\prime },\ldots ,{w_{t_{N}}^{k}}^{\prime }\right)$. The weight of a new word is absent because it is out of the vector space. The short text vector $V_{k}^{\prime }$ feeds the ANN to yield the probabilities $P(ANN\left(V_{k}^{\prime }\right)=ham)$ and $P(ANN\left(V_{k}^{\prime }\right)=spam)$, respectively.

In step 2, the weight of the new word in the *k*th short text is calculated as:

(3a) $$\begin{array}{cc} \; & w_{k}^{new\;word}=\alpha_{k}\times \bar{w^{NW}} \end{array}$$

(3b) $$\begin{array}{cc} \; & \alpha_{k}=\left|P\left(ANN\left(V_{k}^{\prime }\right)=ham\right)-P\left(ANN\left(V_{k}^{\prime }\right)=spam\right)\right| \end{array}$$

where $\bar{w^{NW}}$ is the average value of neighbor words’ weights $w_{1}^{NW},w_{2}^{NW},\ldots ,w_{r}^{NW}$, and *r* is the total number of all neighbor words in the $k^{th}$ short text. Normally, the neighbor words are restricted in the nearest phrases. For the short texts that contain only one phrase, all the pre-processed words are involved in Equations (3a) and (3b).

The weight of a new word in a short text is calculated as the mean of the weights of its neighboring words and adjusted based on the difference between the probabilities. A significant difference suggests that the new word likely contains substantial semantic information related to a specific category, and its weight in the short text is increased. Conversely, if a small difference is observed, it indicates that the new word holds similar semantic importance across different categories, leading to a reduction in its weight. The difference in predicted probabilities, denoted as α, serves as a coefficient to emphasize the weight of the new word, particularly in relation to a single category.

In step 3, the weight for the new word is updated to the average value of its weights in the *q* short texts.
**Algorithm 1** The New Word Weighting Algorithm1:NewWordWeight($V_{1}^{\prime },V_{2}^{\prime },\ldots ,V_{q}^{\prime }$)2:**for** k=1 **to** *q* **do**3:   $P(ANN\left(V_{k}^{\prime }\right)=ham)$, $P\left(ANN\left(V_{k}^{\prime }\right)=spam\right)\leftarrow V_{k}^{\prime }$4:   $\alpha_{k}\leftarrow \left|P\left(ANN\left(V_{k}^{\prime }\right)=ham\right)-P\left(ANN\left(V_{k}^{\prime }\right)=spam\right)\right|$5:   $\bar{w^{NW}}\leftarrow w_{1}^{NW},w_{2}^{NW},\ldots ,w_{r}^{NW}$6:   $w_{k}^{new\;word}\leftarrow \alpha_{k}\times \bar{w^{NW}}$7:**end for**8:$w^{new\;word}\leftarrow \bar{w_{k}^{new\;word}}$, for k=1,2,…,q9:**return** $w^{new\;word}$

**Figure 1:**
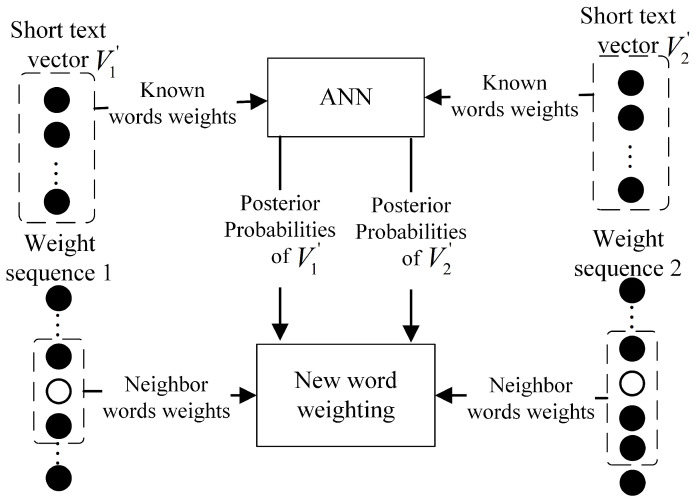
The weighting of a new word occurred in two testing short texts.

For example, Figure 1 shows the weighting process for a new word that occurs in two short texts. Their short text vectors are represented as $V_{1}^{\prime }$ and $V_{2}^{\prime }$ on the top, and the neighbor words sequences in their weight sequences are shown at the bottom. The black dots and circles represent the known words’ weights and the new word weights of the two short texts, respectively. In step 1, the first short text vector $V_{1}^{\prime }$ feeds the ANN, which produces the properties that occurred in the ham and spam categories, and so does $V_{2}^{\prime }$. In step 2, the weights of the new word in the first and second short texts are calculated separately by Equations (3a) and (3b). In step 3, the two weights of the new word are averaged, and the average value is used as the weight of the new word when it appears subsequently in another testing short text.

### 2.4. The HMM for Short Text Filtering

#### 2.4.1. Short Text Representation for the HMM

The HMM is trained by the sequences of word weight and the hidden state pairs, (weight,label). The *i*th short text is pre-processed as a sequence of word weights and can be denoted as $ST_{i}=\left(w_{1}^{ST_{i}},w_{2}^{ST_{i}},w_{3}^{ST_{i}},\ldots ,w_{Length(ST_{i})}^{ST_{i}}\right)$, (0≤i≤M). $w_{j}^{ST_{i}}\in w_{T}$ is the word weight of the $j^{th}$ word of the $i^{th}$ text. The weights set $w_{T}$ is also the set of the observations of the HMM.

In addition, the positive word weights are labeled ham state and the negative word weights are labeled spam state. The short texts are finally transformed into the sequences of the (weight,label) pairs for the supervised training of the HMM.

#### 2.4.2. HMM Formulation and Notation

The parameters of the HMM are a three tuple

(4) λ=(π,A,B),

where π, *A*, and *B* are the initial state distribution, the state transition probability matrix, and the emission probability matrix, respectively.

Because the word weights in short texts are labeled ham or spam state, the hidden states of the HMM can be represented as $S=\left\{s_{1},s_{2}\right\}=\left\{spam,ham\right\}$.

An element of *A* represents the transmission probability between states including self-transmission. $A=\left(a_{ij}\right)\in R^{2\times 2}$, where $a_{ij}=P(q_{t+1}=s_{j}\left|q_{t}=s_{i}\right)$, for i,j=1,2 and $\sum_{j=1}^{2}a_{ij}=1$.

The emission probability matrix, also called the observation probability matrix, $B=(b_{j}\left(x\right))$, for j=1,2, is the outcome of two Gaussian probability distribution functions $b_{j}\left(x\right)=N\left(x,\mu_{j},\sigma_{j}^{2}\right)$, −∞≤x≤∞, where $\mu_{j}$ is the mean of the $j^{th}$ distribution associated with the hidden state $s_{j}$ and $\sigma_{j}$ its standard deviation.

$\pi =(\pi_{1},\pi_{2})$ is the initial probability distribution over the states. $\pi_{1}=P\left(q_{1}=\;s_{1}\right),$ $\pi_{2}\;=P\left(q_{1}=s_{2}\right)$, and $\pi_{1}+\pi_{2}=1$.

#### 2.4.3. Training of the HMM

While training, the parameters λ of the HMM are first initialized as the following: $\pi_{0}=\left[0.5,0.5\right]$ gives the equal opportunity for starting from the ham or spam state. $A_{0}=\left[\begin{array}{cc} 0.5 & 0.5\\ 0.5 & 0.5 \end{array}\right]$ specify the same transmission probability between the two states including self-transmission. $B_{0}$ is initialized by the parameters of the Gaussian probability distribution obtained from the observation sequences, i.e., the word weights sequences, of the spam and ham texts in the training set, separately.

When the training process starts, the (weight,label) pairs sequences feed the HMM for supervised learning. Based on the maximum likelihood estimate method, the transition matrix of *A* can be directly calculated by the relative transition frequency between two states and self-transmission. Also, the Gaussian distribution parameters of the emission matrix *B* can be derived by computing the probabilities of the observation values omitted by a specific hidden state. Thus, the optimal λ is directly computed.

### 2.5. The Proposed Hybrid Model

**Figure 2:**
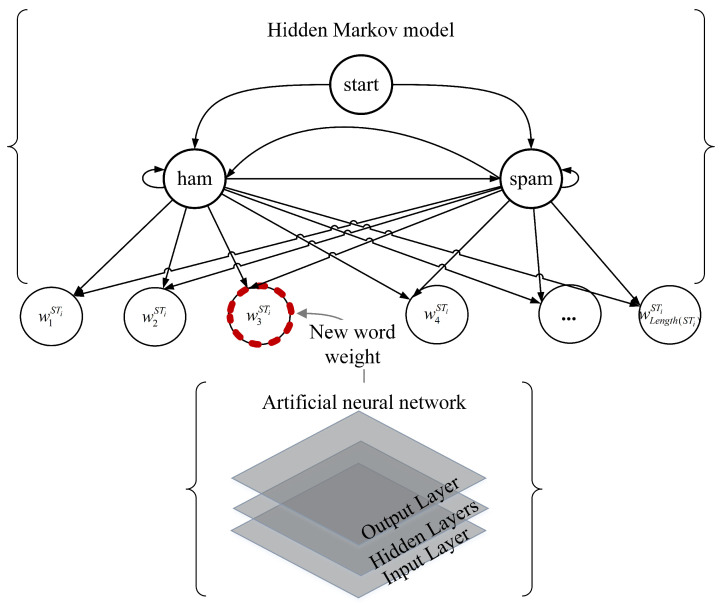
The schematic diagram of the hybrid model.

The schematic diagram of the proposed model is shown in Figure 2, illustrating that the ANN and the HMM are used for new word weighting and spam filtering, respectively.

#### 2.5.1. The Asynchronous Training of the Hybrid Model

**Figure 3:**
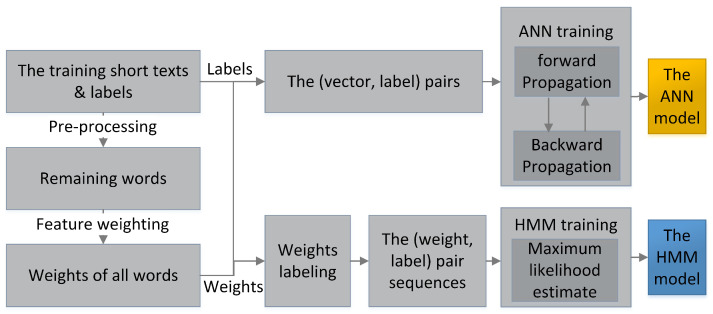
The training process of the hybrid model.

Figure 3 shows the training process of the hybrid model. When the training starts, the short texts in the training set are pre-processed. The remaining words are weighted to form the inputs: the (vector,label) pairs for the ANN and the (weight,label) pair sequences for the HMM. Then, the ANN is trained by the backward propagation algorithm. Meanwhile, (weight,label) pair sequences are joined into one sequence for the HMM batch learning based on the maximum likelihood estimate method.

The HMM can be quickly trained for real-time short text filtering, while the ANN requires slightly longer asynchronous training. The HMM starts filtering as soon as it is ready, reporting unidentified text strings to the ANN when they occur more frequently than the preset threshold *q*. Once the ANN completes its training, it begins new word weighting to assist the HMM filtering. This approach ensures immediate HMM operation and allows for frequent retraining of the ANN with a larger dataset, including new short texts identified by the HMM and manually verified in the production environment. The retrained ANN can incorporate an expanded set of known words with updated weights for more accurate new word weighting.

#### 2.5.2. Filtering with New Word Weighting of the Hybrid Model

**Figure 4:**
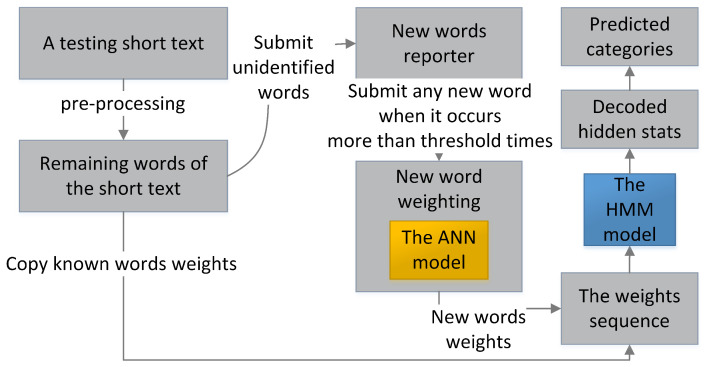
The testing process of the hybrid model.

While filtering, as shown in Figure 4, the testing short texts are individually processed. Each testing short text is first pre-processed and transformed into a weight sequence by copying the weights of the same known words in the training set. Any unidentified text strings in testing short texts are first temperately set at weight 0 for the HMM to fast filter spam and submitted to the new words reporter before being weighted. When the ANN completes training, it calculates the reported new word weight based on the average weights of its neighbor words and the ANN probabilities of all short texts where the new word presents. The new words weights are used when they occur subsequently in other short texts. The HMM decodes the optimal hidden state sequence based on the weight sequence of each testing short text individually. Finally, the short text is classified based on the majority rule.

## 3. Experiments and Results

Experiments are designed to compare the performance of the proposed hybrid model with some well-known reported models in recent studies. The hybrid model is programmed in Python 3.7 with imported packages, such as pomegranate for HMM modeling and sklearn for ANN modeling. The hardware for this experiment is a computer with Intel Core i7-7820 CPU and 16 GB memory (Intel, Santa Clara, CA, USA).

### 3.1. Experiment on the UCI SMS Data Set

First, the benchmark, i.e., the UCI SMS Spam Collection data set, is used for the experiment. This data set is well accepted in SMS filtering research [23]. The SMS messages in the data set are written in English and labeled with either the ham or spam category. The SMS message data set contains 5574 SMS messages, with 747 labeled with spam and 4827 labeled with ham.

The data set is split the same way as other reported models for fair comparison, that is, two-thirds of the messages (3716 messages) are used for training and the remaining messages (1858 messages) are used for testing.

The performance metrics are commonly used in classification problems, including precision (Prec), recall (Rec), F1-measure (F1), accuracy (Acc), and area under the curve (AUC) [6].

### 3.2. Experiment Results and Comparisons on the UCI SMS Data Set

#### 3.2.1. Experiment Results of the HMM

The pre-processing extracts 8127 words from the SMS messages in the training set. The probability distribution parameter π of the trained HMM keeps its initial setting π=[0.5,0.5]. Its transmission matrix is changed to:

(5) $$A=\left[\begin{array}{cc} 0.60178 & 0.39822\\ 0.16348 & 0.83652 \end{array}\right].$$

The first row of Equation (5) indicates the transmission probabilities from the spam state to itself and to the ham state. The second row are the transmission probabilities from the ham state to the spam state and itself. The μ and σ values in the Table 1 are the mean and standard deviation values of the words weights labeled with spam and ham, respectively.

#### 3.2.2. Experiment Results of the ANN

The ANN model underwent 57 training iterations, achieving a final loss of 0.03807. In the testing dataset, comprising 1858 short texts, 249 were categorized as spam and 1609 as ham. When we set the parameter *q* to 1, treating all unknown strings as new words and assigning weights using the ANN, we observed 433 new words in 146 spam testing short texts. Additionally, 1862 new words were detected in 804 ham testing short texts, illustrating the encounter of an extensive vocabulary of new words.

During the experiment, four test messages were identified that did not contain any known words. Two of these were short phrases, “University of Southern California” and “East Coast.” The third message consisted of a list of names: “Mathews, Tait, Edwards, and Anderson.” The fourth message, “Erutupalam thandiyachu,” was written in a non-English language.

#### 3.2.3. Comparisons on the UCI SMS

The confusion matrix is shown in Table 2, indicating a precision value of 99.0% in ham message prediction. The results of other metrics also improved significantly compared with reported works, including NB, SVM, DT, LDA, LSTM, 3CNN, and our two previous works, as shown in the Table 3.

### 3.3. Experiments and Results on Other Data Sets

Extensive binary classification experiments are performed on other datasets, including:

- Dahan: The dataset used in this study is the Dahan SMS spam dataset, containing 14,943 ham messages and 5762 spam messages, in Chinese. These SMS messages were collected in collaboration with our partner company, which operates an enterprise short message service platform handling an average of 150 million short messages daily. The ham messages primarily include notifications from express delivery services, banks, and e-commerce platforms, while the spam messages occasionally originate from registered platform users for advertising purposes.
- MR: A benchmark short text data set of movie reviews [30]. The data set is balanced and contains 5331 positive reviews and 5331 negative reviews, respectively. These movie reviews are processed sentences from the movie reviews published on the website rottentomatoes.com
- CR: A benchmark short text data set of customer reviews [31]. The data set is imbalanced. It includes 2406 positive and 1367 negative reviews for digital products, such as DVD players, MP3 players, and cameras.
- SST-1: A benchmark data set for sentiment analysis, called Stanford sentiment treebank [32]. The data set is extended from MR and is refined with additional labels. In the experiment, the reviews with very positive, positive, negative, and very negative are reserved. The reviews with neutral labels are excluded.

The performance results are shown in Table 4. They are compared with other reported methods in Table 5. The proposed model achieves 0.816, 0.852, and 0.459 accuracy on MR, CR, and SST-1, respectively, which are comparable to the deep learning models. It also achieves a remarkable accuracy of 0.989 on a Chinese SMS data set, showing its capability in multi-language filtering.

### 3.4. Training Time and Throughput Results

Fast filtering is significant in a production environment. We conduct experiments to compare training time and throughput between the proposed hybrid model and deep learning models. The LSTM, 1CNN, and 3CNN models are built, trained, and used for filtering, respectively, against the UCI repository. Each model is stacked with a word embedding layer in front. The dimension of the word-embedding vectors and the fixed length of each short text is set at 50. Short texts are truncated or padded with zeros to that length. Then, for the LSTM model, the number of units is set at 50 matching the length. The 1CNN model includes a one-dimensional convolutional layer, which consists of a collection of 64 kernels of a size 3×3. Similarly, the 3CNN model consists of three of such convolutional layers but with different kernel sizes of 3×3, 4×4, and 5×5, respectively. A max-pooling layer is connected after the convolutional layers. Finally, the models are connected with a dense layer and a softmax classifier in the end [28]. While training, the batch size is set at 50. The LSTM model trains 15 epochs, while the 1CNN and 3CNN models train 20 epochs, before the value of the binary cross entropy loss function decreases below the threshold.

The training time and throughput of the models are shown in Table 6. The training speed of the LSTM, 1CNN and 3CNN models are 76, 720, and 309 SMS messages/s, respectively. The values of their filtering throughput are 286, 14,345, and 7933 messages/s, respectively. None of the filtering speeds can meet the requirement of the production environment [21]. Meanwhile, the proposed model achieves a remarkable training speed of 169,240 messages/s and a filtering throughput of 109,577 messages. The filtering throughput exceeds 34 times the peak SMS filtering throughput requirement of the filtering industry [21].

### 3.5. Discussions

#### 3.5.1. Performance Analysis

The performance is encouraging with the accuracy value of 98.0%. The accuracy surpasses NB, SVM, DT, and LDA because short texts are sequential data and the HMM takes the advantage of word order. In addition, the proposed hybrid model also overtakes some well-known deep learning models, such as LSTM and 3CNN [28]. Furthermore, the hybrid model works better in classifying ham messages, which is critical for the filtering industry. The precision increases greatly to 99.0%.

In our studies, short text classification is regarded as a modeling task for short sequences primarily characterized by short-term dependencies. HMMs prove to be a valuable statistical tool for capturing and modeling these short-term dependencies within a sequence. In our prior works, we introduced two HMM models. The first model, namely, our previous work 1, treated words as sequential discrete observations and achieved an impressive accuracy rate of 95.9% [29]. The second model, our previous work 2, achieved even higher accuracy, with a rate of 96.9% [21], by representing short texts as one-dimensional value sequences. The previous works also revealed that the weight of new words can mislead the HMM decoding process and thus affect classification performance. To address the informal writing issue, the hybrid model uses ANN to calculate weights to new words, while the HMM continues to filter short texts. The sign of the new word weight is determined by the mean of the neighboring word weights, where a positive sign indicates the presence of ham information and a negative sign suggests spam content. Additionally, the magnitude of the new word weight is adjusted proportionally based on the difference between the ANN probabilities for the ham and spam categories. Our experimental results strongly validate the effectiveness of the hybrid method for addressing informal writing issues, demonstrating its ability to enhance the accuracy and throughput of short text classification.

The experiment conducted on the UCI SMS data set has limitations. The precision value for the spam category is 91.2%. Because the UCI data are imbalanced and small; there are only 249 spam messages for testing. Almost no unidentified strings in the spam short messages appear twice and are reported as new words. The model is expected to perform better in large datasets.

The additional experiments on other benchmark datasets also show the effectiveness of the hybrid model. Its accuracy surpasses the majority of the listed outstanding deep learning models, including CNN, Bi-GRU, and LSTM.

#### 3.5.2. Multi-Language Filtering Capabilities

The experiment on the Dahan data set is conducted to validate the multi-language filtering capabilities of the hybrid model. The experiment result of the hybrid model is shown in Table 4. It is confirmed that the hybrid model works better on the Chinese data set. The reason is due to the difference between Chinese and English. Chinese words always have only one form, while English words change their forms according to grammar. In addition, the hybrid model yields better precision in predicting ham messages than spam messages because the malicious messages often has various expressions compared with the legal notifications.

### 3.6. Limitations

Hidden Markov Models are often considered as first-order models, primarily designed to capture short-term dependencies in sequential data. As a result, the method proposed in this study is most effective when applied to tasks like short text classification, where these short-term dependencies play a significant role. When dealing with longer texts or tasks involving longer-term dependencies, alternative models or techniques, designed to capture such relationships, may be more appropriate for achieving accurate results.

## 4. Conclusions and Future Work

A hybrid model that consists of an ANN and an HMM was presented in this paper for spam short text filtering. To handle new words in spam texts, a novel new word weighting approach was developed. The new word weight is related to all short texts where it occurs, and the differences between the ANN prediction probabilities for ham and spam categories are used as the coefficients. The weight of a new word is evaluated based on the weighted mean of the average values of the weights of its neighbor words in the short texts. The hybrid model has been tested against benchmark datasets, including the SMS data set maintained by UCI repository, and it outperforms other outstanding machine learning algorithms. Compared with deep learning models such as CNN and LSTM, the hybrid model achieved a higher accuracy with much faster training speed and filtering throughput. The good balance between accuracy and speed makes it suitable for industrial applications in short text filtering.

This work has confirmed the effectiveness of HMM hybrid models in classifying short texts. Future research will focus on exploring and integrating hybrid methods that combine HMM with other deep learning models.

## Figures and Tables

**Table 1:** The Gaussian distribution parameters of spam and ham states of the emission probabilities matrix *B*.

	μ	σ
Spam	−0.07713	0.35576
Ham	0.19436	0.28185

**Table 2:** The confusion matrix of the hybrid model.

	Actual	Predicted		Percentage%		AUC
		Spam	Ham	Spam	Ham	
Spam	249	227	22	91.2	8.8	0.960
Ham	1609	16	1593	1.0	99.0	

**Table 3:** Comparison of models.

Model	Class	Acc	Prec	Rec	F1
NB [24]	Overall	0.916	0.93	0.92	0.92
SVM [23]	Overall	0.931	0.929	0.931	0.930
DT [25]	Overall	0.965	0.854	0.782	0.816
LDA [26]	Overall	0.904	0.96	0.976	0.92
LSTM [27]	Spam	0.977	0.948	0.901	0.926
	Ham		0.982	0.990	0.986
3CNN [28]	Spam	0.979	0.988	0.858	0.922
	Ham		0.982	0.996	0.988
Our previous	Spam	0.959	0.892	0.816	0.852
work 1 [29]	Ham		0.969	0.983	0.976
Our previous	Spam	0.969	0.936	0.850	0.891
work 2 [21]	Ham		0.975	0.990	0.982
Our	Spam	**0.980**	0.912	0.934	0.923
hybrid model	Ham		**0.990**	0.986	**0.988**

NB: naïve Bayes classifier; SVM: support vector machine; DT: decision tree; LDA: Latent Dirichlet allocation; LSTM: long short-term memory; 3CNN: and convolutional neural networks stacked with three convolutional layers. Bold indicates the highest values.

**Table 4:** Results of the proposed method on MR and CR datasets.

Data Set	Class	Acc	Prec	Rec	F1
Dahan	Spam	0.989	0.983	0.977	0.980
	Ham		0.991	0.993	0.992
MR	Spam	0.816	0.812	0.819	0.815
	Ham		0.821	0.814	0.817
CR	Spam	0.852	0.831	0.770	0.800
	Ham		0.859	0.900	0.879
SST-1	Spam	0.459	0.456	0.459	0.457
	Ham		0.462	0.459	0.460

**Table 5:** Accuracy comparisons with reported models.

Models	MR	CR	SST-1
SVM [33]	0.772	0.795	-
NB [33]	0.737	0.836	-
RCNN [34]	0.816	0.845	-
Bi-GRU [35]	0.815	0.844	-
Convolutional-GRU [36]	0.819	0.850	-
LSTM-att [37]	**0.823**	0.817	0.454
Bi-LSTM-att [38]	0.797	0.814	**0.461**
The proposed hybrid model	0.816	**0.852**	0.459

RCNN: recurrent convolutional neural networks; Bi-GRU: bidirectional gated recurrent unit; Convolutional-GRU: convolutional gated recurrent unit; LSTM-att: Attention-based long short-term memory; and Bi-LSTM-att: bidirectional LSTM with two-dimensional max pooling. Bold indicates the highest values.

**Table 6:** Comparison of training time and throughput.

Models	Training Time (s)	Training Speed (Messages/s) Speed	Filtering Time (s)	Throughput (Messages/s)
LSTM	47.822	76	6.854	286
1CNN	5.022	720	0.136	14,345
3CNN	11.690	309	0.247	7933
The proposed hybrid model	**0.217**	**169,240**	**0.017**	**109,577**

Bold indicates they outperform other values.

## Data Availability

The authors confirm that the data supporting the findings of this study are available from the corresponding author, upon reasonable request.

## References

[1] Al Sulaimani S., Starkey A. (2021). Short Text Classification Using Contextual Analysis. IEEE Access.

[2] Bakr Y., Tolba A., Meshreki H. (2019). Drivers of SMS advertising acceptance: A mixed-methods approach. J. Res. Interact. Mark..

[3] Alsmadi I., Gan K.H. (2019). Review of short-text classification. Int. J. Web Inf. Syst..

[4] Gao Z., Li Z., Luo J., Li X. (2022). Short text aspect-based sentiment analysis based on CNN+ BiGRU. Appl. Sci..

[5] Ghanem R., Erbay H. (2023). Spam detection on social networks using deep contextualized word representation. Multimed. Tools Appl..

[6] Abayomi-Alli O., Misra S., Abayomi-Alli A., Odusami M. (2019). A review of soft techniques for SMS spam classification: Methods, approaches and applications. Eng. Appl. Artif. Intell..

[7] Ruan S., Chen B., Song K., Li H. (2022). Weighted naïve Bayes text classification algorithm based on improved distance correlation coefficient. Neural Comput. Appl..

[8] Samant S.S., Murthy N.L.B., Malapati A. (2019). Improving Term Weighting Schemes for Short Text Classification in Vector Space Model. IEEE Access.

[9] Dang E.K.F., Luk R.W.P., Allan J. (2020). Context-dependent feature values in text categorization. Int. J. Softw. Eng. Knowl. Eng..

[10] Oyelade O.N., Agushaka J.O., Ezugwu A.E. (2023). Evolutionary binary feature selection using adaptive ebola optimization search algorithm for high-dimensional datasets. PLoS ONE.

[11] Bansal B., Srivastava S. (2019). Hybrid attribute based sentiment classification of online reviews for consumer intelligence. Appl. Intell..

[12] Bello A., Ng S.C., Leung M.F. (2023). A BERT framework to sentiment analysis of tweets. Sensors.

[13] Machicao J., Corrêa E.A., Miranda G.H., Amancio D.R., Bruno O.M. (2018). Authorship attribution based on life-like network automata. PLoS ONE.

[14] Ghourabi A., Alohaly M. (2023). Enhancing Spam Message Classification and Detection Using Transformer-Based Embedding and Ensemble Learning. Sensors.

[15] Liao W., Zeng B., Yin X., Wei P. (2021). An improved aspect-category sentiment analysis model for text sentiment analysis based on RoBERTa. Appl. Intell..

[16] Wang H., Tian K., Wu Z., Wang L. (2021). A Short Text Classification Method Based on Convolutional Neural Network and Semantic Extension. Int. J. Comput. Intell. Syst..

[17] Cai T., Zhang X. (2023). Imbalanced Text Sentiment Classification Based on Multi-Channel BLTCN-BLSTM Self-Attention. Sensors.

[18] Abid M.A., Ullah S., Siddique M.A., Mushtaq M.F., Aljedaani W., Rustam F. (2022). Spam SMS filtering based on text features and supervised machine learning techniques. Multimed. Tools Appl..

[19] Qian Y., Du Y., Deng X., Ma B., Ye Q., Yuan H. (2019). Detecting new Chinese words from massive domain texts with word embedding. J. Inf. Sci..

[20] Duan J., Tan Z., Zhang M., Wang H. (2020). New word detection using BiLSTM+CRF model with features. IEICE Trans. Inf. Syst..

[21] Xia T., Chen X. (2021). A weighted feature enhanced Hidden Markov Model for spam SMS filtering. Neurocomputing.

[22] Salton G., Wong A., Yang C.S. (1975). A vector space model for automatic indexing. Commun. ACM.

[23] Jain G., Sharma M., Agarwal B. (2019). Spam detection in social media using convolutional and long short term memory neural network. Ann. Math. Artif. Intell..

[24] Mishra S., Soni D. (2020). Smishing Detector: A security model to detect smishing through SMS content analysis and URL behavior analysis. Future Gener. Comput. Syst..

[25] Ghourabi A., Mahmood M.A., Alzubi Q.M. (2020). A hybrid CNN-LSTM model for SMS spam detection in arabic and english messages. Future Internet.

[26] Nagwani N.K., Sharaff A. (2017). SMS spam filtering and thread identification using bi-level text classification and clustering techniques. J. Inf. Sci..

[27] Shaaban M.A., Hassan Y.F., Guirguis S.K. (2022). Deep convolutional forest: A dynamic deep ensemble approach for spam detection in text. Complex Intell. Syst..

[28] Roy P.K., Singh J.P., Banerjee S. (2020). Deep learning to filter SMS Spam. Future Gener. Comput. Syst..

[29] Xia T., Chen X. (2020). A discrete hidden Markov model for SMS spam detection. Appl. Sci..

[30] Pang B., Lee L. (2005). Seeing stars: Exploiting class relationships for sentiment categorization with respect to rating scales. arXiv.

[31] Hu M., Liu B. Mining and summarizing customer reviews. Proceedings of the Tenth ACM SIGKDD International Conference on Knowledge Discovery and Data Mining.

[32] Socher R., Perelygin A., Wu J., Chuang J., Manning C.D., Ng A.Y., Potts C. Recursive deep models for semantic compositionality over a sentiment treebank. Proceedings of the 2013 Conference on Empirical Methods in Natural Language Processing.

[33] Liu Z., Kan H., Zhang T., Li Y. (2020). DUKMSVM: A framework of deep uniform kernel mapping support vector machine for short text classification. Appl. Sci..

[34] Wang R., Li Z., Cao J., Chen T., Wang L. Convolutional recurrent neural networks for text classification. Proceedings of the 2019 International Joint Conference on Neural Networks (IJCNN).

[35] Cheng Y., Yao L., Xiang G., Zhang G., Tang T., Zhong L. (2020). Text Sentiment Orientation Analysis Based on Multi-Channel CNN and Bidirectional GRU with Attention Mechanism. IEEE Access.

[36] Zhang Z., Robinson D., Tepper J. Detecting Hate Speech on Twitter Using a Convolution-GRU Based Deep Neural Network. Proceedings of the 15th Semantic Web International Conference.

[37] Wang Y., Huang M., Zhao L., Zhu X. Attention-based LSTM for aspect-level sentiment classification. Proceedings of the EMNLP 2016—Conference on Empirical Methods in Natural Language Processing.

[38] Zhou P., Qi Z., Zheng S., Xu J., Bao H., Xu B. (2016). Text classification improved by integrating bidirectional LSTM with two-dimensional max pooling. arXiv.

